# Heart Failure Etiology and 6-Month Cardiorenal Recovery Patterns After Early In-Hospital SGLT2 Inhibitor Initiation in HFrEF: A Prospective Real-World Cohort

**DOI:** 10.3390/medicina62061017

**Published:** 2026-05-24

**Authors:** Marija Radić, Ivana Jurin, Fran Rode, Luka Šimunović, Petra Kolundžić, Irzal Hadžibegović, Šime Manola, Petra Vitlov, Vanja Ivanović Mihajlović, Danijela Grizelj, Hrvoje Falak, Mario Udovičić, Tomislav Letilović

**Affiliations:** 1 Department of Cardiovascular Diseases, University hospital Dubrava, 10000 Zagreb, Croatia; ivanajurin1912@gmail.com (I.J.); irzalh@gmail.com (I.H.); sime.manola@icloud.com (Š.M.); petra.vitlov@gmail.com (P.V.); vanja42@gmail.com (V.I.M.); danijela.grizelj@yahoo.com (D.G.); hrvoje.falak@gmail.com (H.F.); mario.udovicic@gmail.com (M.U.); 2 Department of Orthodontics, School of Dental Medicine, University of Zagreb, Gundulićeva 5, 10000 Zagreb, Croatia; lsimunovic@sfzg.hr; 3 School of Medicine, University of Zagreb, 10000 Zagreb, Croatia; petra.kolundzic@gmail.com (P.K.); tomislavletilovic@gmail.com (T.L.); 4 Faculty of Dental Medicine and Health Care, Josip Juraj Strossmayer University of Osijek, 31000 Osijek, Croatia; 5 Department of Cardiology, University Hospital Merkur, Zajceva 19, 10000 Zagreb, Croatia

**Keywords:** heart failure with reduced ejection fraction, sodium–glucose cotransporter 2 inhibitors, ischemic cardiomyopathy, non-ischemic cardiomyopathy, reverse remodeling, renal function, NT-proBNP, real-world cohort

## Abstract

*Background*: SGLT2 inhibitors improve outcomes in heart failure with reduced ejection fraction (HFrEF), but whether early recovery patterns after initiation differ according to HF etiology in real-world practice remains uncertain. *Objective*: To evaluate whether ischemic versus non-ischemic etiology is associated with different 6-month cardiac, renal, biomarker, and exploratory metabolic trajectories after early in-hospital SGLT2 inhibitor initiation in HFrEF. *Materials and Methods*: In this prospective single-center observational cohort (2022–2025), consecutive adults hospitalized with first-presentation acute HFrEF who initiated empagliflozin or dapagliflozin within 48 h of admission were enrolled. Patients were classified as having ischemic cardiomyopathy (ICM) or non-ischemic cardiomyopathy (NICM). The primary analytic cohort included patients with paired baseline and 6-month echocardiography. The primary outcome was change in left ventricular ejection fraction (LVEF); eGFR and NT-proBNP were secondary outcomes. Exploratory metabolic/laboratory variables were summarized descriptively using paired available-case follow-up. The study was approved by the institutional ethics committee and registered in ClinicalTrials.gov under the CaRD registry framework (NCT06090591). *Results*: The paired 6-month echocardiographic analytic cohort comprised 241 patients who survived to reassessment (ICM *n* = 90; NICM *n* = 151). NICM showed greater improvement in LVEF than ICM (ΔLVEF +10% [IQR 0–18] vs. +5% [IQR 0–12]; *p* = 0.049) and a more favorable eGFR trajectory (ΔeGFR 0.30 [IQR −5.90 to 6.60] vs. −2.70 [IQR −12.60 to 3.40] mL/min/1.73 m^2^; *p* = 0.038). NT-proBNP declined substantially in both groups, with no between-group difference in change magnitude (*p* = 0.845), although 6-month values remained higher in ICM (*p* = 0.034). However, after multivariable adjustment, ischemic etiology was no longer independently associated with 6-month LVEF or eGFR outcomes. Exploratory metabolic findings varied descriptively by etiology but should be interpreted cautiously because follow-up completeness and background treatment intensity varied across variables. *Conclusions*: In this real-world cohort of patients with HFrEF who initiated SGLT2 inhibitors during hospitalization, HF etiology was associated with different short-term cardiorenal recovery patterns, whereas NT-proBNP reduction was similar across groups. These findings characterize etiology-related recovery within a treated cohort rather than differential SGLT2 inhibitor efficacy and should therefore be considered as hypothesis-generating.

## 1. Introduction

Heart failure (HF) is a clinically heterogeneous syndrome arising from diverse structural and functional cardiac disorders. In HFrEF, etiologic classification is not merely descriptive; it influences prognosis, likelihood of reverse remodeling, and interpretation of early changes after treatment initiation.

Within HFrEF, ischemic cardiomyopathy (ICM) and non-ischemic cardiomyopathy (NICM) represent two biologically distinct entities with different underlying pathophysiology. ICM results from atherosclerotic coronary artery disease leading to myocardial ischemia, infarction, and subsequent replacement fibrosis, resulting in regionally heterogeneous contractile dysfunction. The resulting dysfunction is frequently irreversible in areas of established myocardial scar, which constrains the potential for recovery [1]. In contrast, non-ischemic cardiomyopathy (NICM) encompasses a diverse group of non-atherosclerotic disorders—including genetic, inflammatory, toxic, and idiopathic forms—characterized more often by diffuse myocardial involvement, cellular dysfunction and interstitial fibrosis without significant epicardial coronary obstruction, and generally associated with greater potential for reversibility of myocardial dysfunction [2]. These pathophysiological differences are clinically relevant, as ICM remains one of the leading causes of HFrEF globally, while NICM represents a heterogeneous but substantial proportion of the remaining burden.

Data from the REPORT-HF registry indicate that coronary artery disease remains common among patients hospitalized with acute HF, underscoring the continuing clinical relevance of ischemic etiology in contemporary practice [3]. Given these fundamental differences in myocardial substrate between ICM and NICM, whether response to SGLT2 inhibitor initiation differs across etiologic subgroups is clinically important.

Implementation of guideline-directed medical therapy has substantially improved outcomes in HFrEF. SGLT2 inhibitors are now a foundational component of contemporary treatment, and hospitalization offers an important window for early initiation alongside broader optimization of disease-modifying therapy [4,5,6,7]. However, early treatment effects may not be uniform across etiologies, given differences in myocardial substrate, scar burden, and cardiorenal vulnerability. Clarifying these early trajectories across etiologic subgroups therefore represents an important and insufficiently explored aspect of contemporary HFrEF management.

Randomized trial populations are selected, whereas real-world HFrEF cohorts are more heterogeneous with respect to age, comorbidity burden, disease severity, background therapy, and underlying substrate. Most analyses of SGLT2 inhibitor therapy have emphasized ejection-fraction phenotype, diabetes status, or renal function, with comparatively little attention to HF etiology as a potential determinant of early recovery patterns [8,9].

This distinction is clinically relevant because ischemic scar burden may constrain reverse remodeling, while concomitant atherosclerotic and renovascular disease may increase cardiorenal vulnerability. Accordingly, early trajectories after SGLT2 inhibitor initiation may differ across etiologic subgroups. However, direct real-world comparisons of cardiac remodeling, renal course, and biomarker response between ICM and NICM after in-hospital initiation remain limited.

We therefore conducted a prospective real-world cohort study to evaluate whether HF etiology was associated with 6-month echocardiographic, renal, biomarker, and exploratory metabolic trajectories after early in-hospital SGLT2 inhibitor initiation in patients with HFrEF.

## 2. Materials and Methods

### 2.1. Study Design and Setting

This prospective, single-center observational cohort study was conducted at a tertiary care center between 2022 and 2025. Consecutive adults hospitalized with a first presentation of acute HFrEF who initiated an SGLT2 inhibitor within 48 h of admission were enrolled and prospectively followed. Enrollment included all eligible patients hospitalized during the study period who fulfilled inclusion criteria and underwent early in-hospital initiation of empagliflozin or dapagliflozin according to routine clinical practice. HF was diagnosed on the basis of compatible symptoms and signs, elevated natriuretic peptides, and imaging evidence of cardiac dysfunction, consistent with routine clinical practice and the Framingham criteria [10].

### 2.2. Ethics

The study was conducted in accordance with the Declaration of Helsinki and was approved by the Ethics Committee of University Hospital Dubrava, Zagreb, Croatia (Approval No. 2022/1403-01; approved on 14 March 2022). The study was registered at ClinicalTrials.gov (NCT06090591), an institutional umbrella registry encompassing multiple prospective observational cardiovascular cohorts conducted within the Department of Cardiology. Although public registry registration was completed subsequently, institutional ethics approval had been obtained before initiation of this observational registry-based cohort study. The study reflected routine clinical practice and initiation of SGLT2 inhibitor therapy was determined by the treating physicians rather than assigned by the study protocol. Accordingly, the study was not originally considered to require prospective registration as an interventional clinical trial before participant enrollment.

All participants provided written informed consent in accordance with institutional requirements.

### 2.3. Study Population

Patients were eligible if they were ≥18 years of age, had a first hospitalization for acute HFrEF defined by symptoms and signs of HF, elevated natriuretic peptides, and objective evidence of cardiac dysfunction on imaging, and initiated empagliflozin or dapagliflozin during the index hospitalization. For inclusion in the paired trajectory analyses, baseline and 6-month follow-up transthoracic echocardiography (TTE) had to be available for assessment of change in left ventricular ejection fraction (LVEF). Secondary and exploratory laboratory analyses were performed using paired available-case data within this echocardiographic cohort; accordingly, effective sample size varied according to endpoint.

Patients were excluded if they had a known allergy, intolerance, or contraindication to SGLT2 inhibitors; pericardial disease; infiltrative cardiomyopathy (including cardiac amyloidosis); hypertrophic cardiomyopathy; type 1 diabetes mellitus; clinically significant primary pulmonary disease; active malignancy under treatment; active systemic autoimmune disease or infection (excluded in their active phase only, not quiescent disease); moderate to severe hepatic dysfunction (Child-Pugh B or C), or elevated transaminases (ALT/AST >3× upper limit of normal); or severe renal impairment (estimated glomerular filtration rate [eGFR] < 30 mL/min/1.73 m^2^).

These exclusions were applied to minimize confounding from conditions not primarily related to HFrEF pathophysiology, or with distinct disease trajectories, or comorbidities that could independently influence cardiac remodeling, renal function or biomarker dynamics, as well as to ensure the safety and interpretability of SGLT2 inhibitor initiation.

Notably, only active systemic autoimmune disease was excluded; patients with quiescent or prior disease were eligible, as the primary concern relates to active systemic inflammation and its effect on vascular tone, inflammatory biomarkers, and fluid regulation.

### 2.4. Etiology Definition

HF etiology was classified as ICM or NICM on the basis of clinical history and available diagnostic testing. ICM required left ventricular systolic dysfunction in the presence of coronary disease judged sufficient to account for the cardiomyopathy, including prior myocardial infarction, prior coronary revascularization, angiographically significant obstructive coronary artery disease, and/or ischemic scar on cardiac magnetic resonance imaging.

NICM was defined as left ventricular systolic dysfunction without prior myocardial infarction or coronary revascularization and without evidence of clinically significant obstructive coronary artery disease on available diagnostic data; non-atherosclerotic etiologies such as hypertensive heart disease, dilated cardiomyopathy, valvular heart disease, tachycardia-induced cardiomyopathy, post-myocarditis cardiomyopathy, and toxic cardiomyopathy were included in this category.

### 2.5. Data Collection and Measurements

Baseline clinical characteristics, comorbidities, and New York Heart Association (NYHA) functional class were recorded during the index hospitalization. Laboratory assessment at baseline and 6 months included serum creatinine, urea, glucose, lipid profile, albumin, hemoglobin/hematocrit, HbA1c, C-reactive protein, and NT-proBNP, where available as part of routine care. Exploratory laboratory and metabolic variables therefore reflected routine follow-up rather than a rigid sampling protocol. Renal function was expressed as eGFR (mL/min/1.73 m^2^).

Transthoracic echocardiography was performed at baseline and at 6 months as part of routine clinical care. LVEF was assessed using standard echocardiographic methods predominantly by Simpson biplane methodology when image quality permitted, in accordance with routine laboratory practice, in accordance with institutional echocardiographic protocols. Follow-up echocardiography was performed using the same routine clinical imaging approach as at baseline. Given the observational real-world design, echocardiographic interpretation was not formally blinded to HF etiology and interobserver variability was not prospectively assessed.

### 2.6. Outcomes

The primary outcome was change in LVEF from baseline to 6 months. Secondary outcomes were changes in eGFR and NT-proBNP. Exploratory outcomes included selected metabolic and laboratory variables collected during routine follow-up, including lipid fractions, HbA1c, and body mass index. These variables were analyzed descriptively and were intended to provide contextual information rather than serve as formal comparative efficacy endpoints.

### 2.7. Sample Size

As this was a consecutive-enrollment observational cohort, no formal recruitment target or power-based stopping rule was prespecified. The final sample therefore reflects all eligible patients accrued during the study period, and between-group comparisons should be interpreted as pragmatic and hypothesis-generating.

### 2.8. Statistical Analysis

Continuous variables were assessed for normality using the Shapiro–Wilk test and visual inspection of histograms and Q-Q plots. Normally distributed variables are presented as mean ± standard deviation (SD) and were compared with Student’s *t*-test; non-normally distributed variables are presented as median (interquartile range [IQR]) and were compared with the Mann–Whitney U test. Categorical variables are presented as counts (percentages) and were compared with the χ^2^ test or Fisher’s exact test, as appropriate.

Multivariable linear regression analyses using an ANCOVA-style approach were performed for 6-month LVEF, eGFR, and NT-proBNP outcomes. Each model included the baseline value of the respective outcome, age, sex, diabetes mellitus, baseline eGFR, baseline NT-proBNP, and key guideline-directed medical therapy variables (ACEi/ARB/ARNI, beta-blocker, and mineralocorticoid receptor antagonist therapy), with HF etiology included as the principal predictor variable. Because of skewed distribution, NT-proBNP values were natural log-transformed before analysis.

Change over time was defined as the 6-month value minus baseline value (Δ). The principal inferential analyses consisted of between-group comparisons of paired change scores using Student’s *t*-test or the Mann–Whitney U test, as appropriate. For laboratory outcomes obtained in routine care, paired analyses were performed using available-case data for each specific endpoint. Endpoint-specific follow-up completeness varied across exploratory laboratory variables because reassessment reflected routine clinical practice rather than a protocolized follow-up schedule.

The primary comparison was the between-group difference in ΔLVEF within the paired echocardiographic cohort. Prespecified secondary comparisons included ΔeGFR and ΔNT-proBNP, whereas metabolic and other laboratory analyses were exploratory and descriptive. Effect sizes for between-group comparisons of paired change scores were additionally reported as Hodges–Lehmann median differences with corresponding 95% confidence intervals.

Given the observational design, baseline group differences, survivor conditioning of the analytic cohort, and endpoint-specific follow-up completeness, the findings should be interpreted cautiously as observational associations within a treated cohort rather than estimates of causal treatment effect.

No imputation or formal multiplicity adjustment was applied because the study was observational and hypothesis-generating.

All statistical tests were two-sided, and *p* < 0.05 was considered statistically significant.

## 3. Results

### 3.1. Participant Characteristics

**Figure 1:**
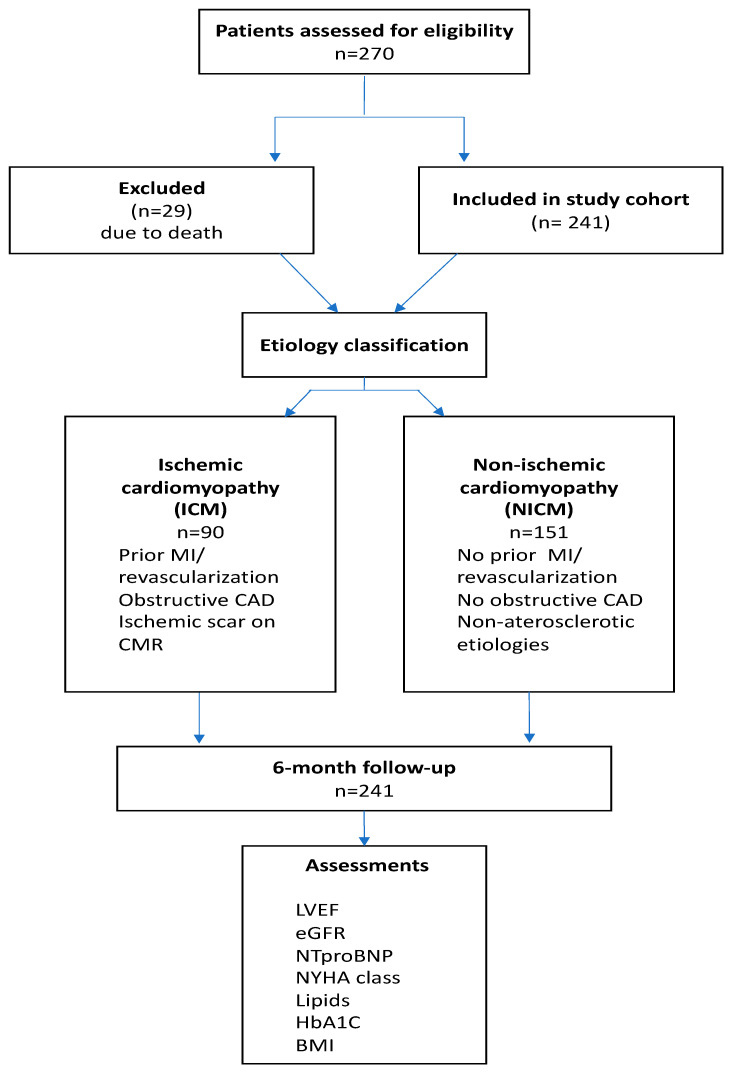
Study flow diagram showing patient screening, eligibility assessment, exclusion criteria, and derivation of the final paired 6-month survivor cohort used for trajectory analyses. Of 270 patients hospitalized with first-presentation acute HFrEF, 29 died before planned reassessment, leaving 241 patients with paired baseline and 6-month echocardiographic follow-up (ICM *n* = 90; NICM *n* = 151). HFrEF: heart failure with reduced ejection fraction; ICM: ischemic cardiomyopathy; NICM: non-ischemic cardiomyopathy.

Among 270 patients hospitalized with first-presentation acute HFrEF during the study period, 29 died before the planned 6-month reassessment and therefore could not contribute to paired echocardiographic trajectory analyses. The final analytic cohort therefore comprised 241 survivors with paired follow-up data, stratified into ICM (*n* = 90) and NICM (*n* = 151) (Figure 1). Baseline characteristics are summarized in Table 1. Patients with ICM were older than those with NICM (64.03 ± 11.17 vs. 60.40 ± 14.01 years, *p* = 0.026). NYHA functional class at admission was similar between groups, with most patients classified as NYHA III (ICM 44.4% vs. NICM 50.9%; *p* = 0.716). Coronary angiography was performed in 95.6% of patients in the ischemic cardiomyopathy group and in 75.5% of patients in the non-ischemic cardiomyopathy group, whereas 4.4% and 24.5% of patients, respectively, did not undergo invasive coronary assessment. The difference in angiography utilization between groups was statistically significant (χ^2^ = 20.800; *p* < 0.001). Additional etiologic evaluation, including cardiac magnetic resonance imaging, coronary CT angiography, or stress testing, was performed when clinically indicated according to routine practice. Arterial hypertension was more prevalent in ICM than in NICM (80.7% vs. 65.1%, *p* = 0.006), and active smoking was also more common in ICM (52.6% vs. 35.8%, *p* = 0.010). Hyperlipidemia was markedly more frequent in ICM (84.4% vs. 50.9%; *p* < 0.001), consistent with a greater burden of atherosclerotic comorbidity. Baseline urea, creatinine, and eGFR values did not significantly differ between the ICM and NICM groups (*p* = 0.552, *p* = 0.815, and *p* = 0.435, respectively), suggesting broadly comparable renal function at admission. Although median baseline LVEF was 30.0% in both groups, the distribution was modestly higher in ICM (IQR 25.0–40.0 vs. 22.0–35.0; *p* = 0.006). Admission glucose levels were also higher in ICM (median 7.25 mmol/L [IQR 6.00–10.90] vs. 6.40 mmol/L [IQR 5.20–7.40]; *p* < 0.001).

### 3.2. Changes over Time

**Figure 2:**
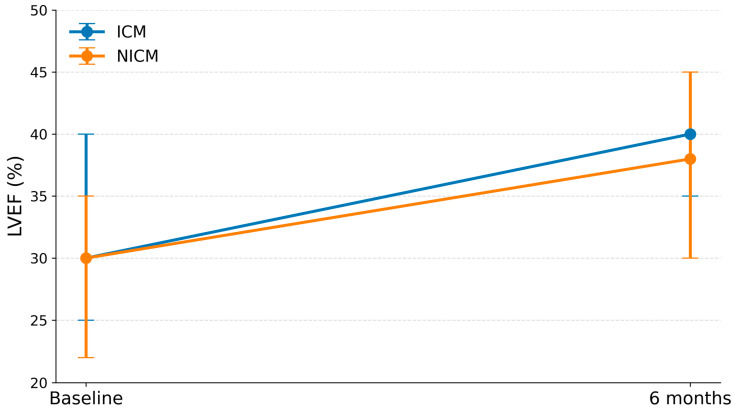
Median left ventricular ejection fraction (LVEF) at baseline and 6-month follow-up according to heart failure etiology. Patients with non-ischemic cardiomyopathy (NICM) demonstrated greater improvement in LVEF over time compared with those with ischemic cardiomyopathy (ICM). Lines represent median values and error bars indicate interquartile ranges (IQRs). Between-group comparison of paired ΔLVEF change scores: *p* = 0.049.

**Figure 3:**
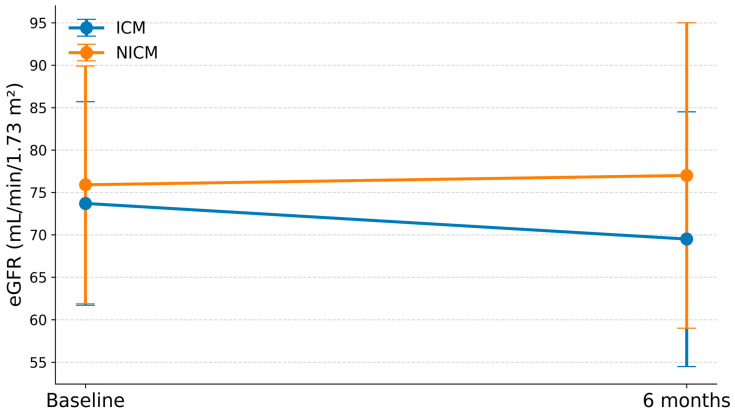
Median estimated glomerular filtration rate (eGFR) at baseline and 6-month follow-up according to heart failure etiology. Patients with NICM showed a more favorable renal trajectory over 6 months compared with ICM. Values are presented as median with interquartile ranges (IQRs). Between-group comparison of paired ΔeGFR change scores: *p* = 0.038.

**Figure 4:**
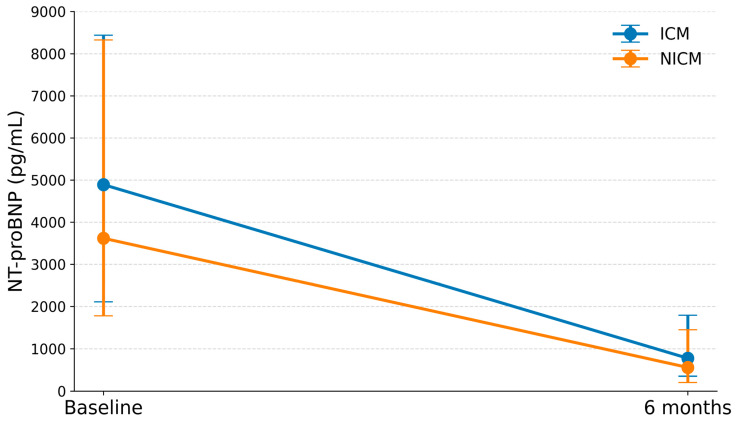
Median NT-proBNP values at baseline and 6-month follow-up according to heart failure etiology. NT-proBNP levels decreased substantially in both ischemic and non-ischemic cardiomyopathy groups, without a significant between-group difference in magnitude of change. Values are presented as median with interquartile ranges [IQRs]. Between-group comparison of paired ΔNT-proBNP change scores: *p* = 0.845.

Unadjusted between-group comparisons of paired change scores within this survivor cohort are summarized in Table 2 and Figure 2, Figure 3 and Figure 4. Because laboratory follow-up reflected routine care rather than a prespecified sampling protocol, endpoint-specific sample sizes varied across exploratory laboratory analyses according to follow-up availability.

For the primary endpoint, NICM showed a greater median improvement in LVEF than ICM: ΔLVEF +10% [IQR 0–18] versus +5% [IQR 0–12], respectively. The between-group comparison reached nominal statistical significance (*p* = 0.049). The Hodges–Lehmann median difference in paired change scores, expressed as NICM minus ICM, was +2.0 percentage points, with a 95% confidence interval of 0.0 to 5.0 percentage points.

A similar borderline pattern was observed for renal trajectory. Median ΔeGFR was +0.30 mL/min/1.73 m^2^ [IQR −5.90 to 6.60] in NICM and −2.70 mL/min/1.73 m^2^ [IQR −12.60 to 3.40] in ICM. The Hodges–Lehmann median difference in paired change scores, expressed as NICM minus ICM, was +3.5 mL/min/1.73 m^2^, with a 95% confidence interval of 0.2 to 6.9 mL/min/1.73 m^2^. The nominal between-group comparison was statistically significant (*p* = 0.038), although the lower confidence bound was close to the null value.

NT-proBNP declined substantially in both groups. The magnitude of change did not differ between NICM and ICM (*p* = 0.845), although absolute 6-month NT-proBNP remained higher in ICM (*p* = 0.034). Baseline, follow-up, and paired change values of key cardiorenal biomarkers are summarized in Table 3.

NYHA functional class at 6 months was similar between groups, with most patients classified as NYHA I and no significant between-group difference (χ^2^ = 0.852, *p* = 0.653).

Exploratory metabolic findings also appeared to vary by etiology. Patients with ICM showed larger reductions in total and LDL cholesterol (both *p* < 0.001), whereas HDL cholesterol increased modestly in NICM (*p* = 0.005). BMI declined in both groups, with a greater reduction in ICM (*p* = 0.035). These findings should be interpreted cautiously because follow-up completeness, concomitant lipid-lowering therapy, and background risk profiles likely differed by etiology.

### 3.3. Multivariable Analyses

Multivariable linear regression analyses were performed to evaluate the independent association between ischemic etiology and 6-month LVEF, eGFR, and NT-proBNP values. Models were adjusted for baseline LVEF, baseline eGFR, baseline log-transformed NT-proBNP, age, sex, diabetes mellitus, ischemic versus non-ischemic etiology, prior ACE inhibitor/ARB/ARNI therapy, prior beta-blocker therapy, and mineralocorticoid receptor antagonist use. For 6-month LVEF, the overall model was significant (F = 7.803, *p* < 0.001; adjusted R^2^ = 0.240). After adjustment, ischemic etiology was not independently associated with follow-up LVEF (B = −1.971, *p* = 0.116), whereas baseline LVEF remained the strongest independent predictor of 6-month LVEF (B = 0.600, *p* < 0.001). For 6-month eGFR, the overall model was significant (F = 43.918, *p* < 0.001; adjusted R^2^ = 0.668). Ischemic etiology was not independently associated with follow-up eGFR after adjustment (B = −3.109, *p* = 0.117). Baseline eGFR was the strongest predictor of 6-month renal function (B = 0.761, *p* < 0.001), while older age was independently associated with lower follow-up eGFR (B = −0.325, *p* < 0.001). For 6-month NT-proBNP, the overall model was also significant (F = 13.164, *p* < 0.001; adjusted R^2^ = 0.366). After adjustment, ischemic etiology was not independently associated with 6-month NT-proBNP levels (B = 0.279, *p* = 0.058). Baseline log-transformed NT-proBNP values remained independently associated with follow-up levels (B = 0.402, *p* < 0.001). Regression diagnostics demonstrated no substantial deviations from linear regression assumptions.

## 4. Discussion

The principal message of this prospective real-world cohort is not that SGLT2 inhibitors can be shown here to have different causal effects by etiology, but rather that observable recovery trajectories after early in-hospital initiation appear to remain conditioned by underlying myocardial and cardiorenal substrate. The present findings should be interpreted within the context of an observational real-world cohort reflecting routine clinical care rather than a protocol-driven interventional study design.

Compared with ICM, patients with NICM showed greater improvement in LVEF and a more favorable net renal trajectory over 6 months, whereas NT-proBNP fell substantially and to a similar extent in both groups. Framed cautiously, the data suggest that the observable phenotype of early recovery remains conditioned by myocardial substrate and cardiorenal vulnerability even when all patients receive the same therapeutic class.

The larger improvement in LVEF observed in NICM is biologically credible. Non-ischemic disease often includes dysfunctional but potentially recoverable myocardium, so relief of congestion, afterload, and neurohormonal activation may translate into more visible reverse remodeling. By contrast, ICM more commonly involves fixed scar, regional contractile loss, and chronic adverse remodeling, which can limit absolute improvement in LVEF even when symptoms and natriuretic peptides improve [1,2]. At the same time, NICM started from a slightly lower baseline LVEF distribution, leaving more room for absolute gain and raising the possibility of regression to the mean. The observation that admission glucose was higher in ICM despite only modest differences in HbA1c may reflect greater stress-related neurohormonal and inflammatory activation during acute decompensation rather than markedly different chronic glycemic exposure.

Because scar burden, revascularization status, and time-updated guideline-directed medical therapy (GDMT) intensification during follow-up were not systematically characterized, these findings should be interpreted as association between myocardial substrate and observed recovery rather than evidence of differential SGLT2 inhibitor efficacy.

The renal findings merit equal caution. SGLT2 inhibitors have established cardiorenal actions, including modulation of intraglomerular pressure, natriuresis, and improved tubular energetics and oxygen handling [11,12,13]. Emerging evidence also suggests broader pleiotropic cardiovascular effects of SGLT2 inhibitors beyond glycemic and renal mechanisms, including favorable effects on arrhythmic burden and myocardial remodeling [14].

Yet renal course in recently hospitalized HFrEF is also shaped by age, diabetes burden, vascular disease, diuretic exposure, renal reserve, and hemodynamic stability. Because only baseline and 6-month creatinine values were examined, the analysis captures the net renal trajectory and cannot distinguish an expected early hemodynamic eGFR dip from later stabilization. The less favorable eGFR course in ICM therefore most likely reflects the joint influence of treatment, substrate, and greater cardiorenal vulnerability, not a simple etiologic pharmacodynamic difference.

By contrast, NT-proBNP fell markedly in both groups, and the magnitude of reduction was similar. This pattern is clinically informative because it suggests that early unloading and decongestive benefit may be achievable across etiologies even when structural recovery and renal evolution diverge. Biomarker response, reverse remodeling, and renal course should therefore not be treated as interchangeable surrogates of improvement or as a single universal proxy of response. Persistently higher 6-month NT-proBNP in ICM may reflect greater residual scar burden, ongoing ischemic substrate, or higher residual filling stress despite overall clinical improvement [15].

The exploratory metabolic findings warrant especially restrained interpretation. Larger reductions in total and LDL cholesterol in ICM are far more plausibly explained by secondary prevention intensity, particularly more frequent or more intensive lipid-lowering therapy in patients with established CAD, than by a direct lipid effect of SGLT2 inhibition itself [16]. Likewise, changes in BMI and other laboratory markers likely reflect a mixture of decongestion, nutritional change, disease recovery, and concomitant treatment optimization. Because follow-up completeness for these exploratory variables reflected routine care, HbA1c and other laboratory measures were not emphasized as formal comparative endpoints. These measures are therefore best viewed as contextual descriptors rather than mechanistic endpoints.

Overall, the study supports an etiology-aware and multiparametric approach to early follow-up after SGLT2 inhibitor initiation in HFrEF. A favorable fall in NT-proBNP should not be assumed to imply equivalent reverse-remodeling potential across etiologies, and a larger increase in LVEF should be interpreted alongside baseline severity, renal course, and time-updated background therapy. Although differences in ΔLVEF and ΔeGFR reached nominal statistical significance, these findings should be interpreted cautiously given the observational design, unequal group sizes, absence of multiplicity adjustment, and confidence intervals approaching the null. Importantly, in additional multivariable analyses adjusting for baseline clinical characteristics, renal function, baseline LVEF, NT-proBNP, and background medical therapy, ischemic etiology was no longer independently associated with 6-month LVEF, eGFR, or NT-proBNP values. These findings suggest that part of the observed between-group differences in unadjusted analyses may reflect baseline imbalance and residual confounding rather than an independent etiologic effect. In addition, although exploratory multivariable models incorporated major cardiorenal and treatment-related covariates, residual confounding related to incompletely modeled baseline clinical heterogeneity cannot be excluded. Several clinically relevant baseline differences, including hypertension, smoking status, hyperlipidemia, and admission glucose, were not included in the exploratory models in order to preserve model stability within the available sample size.

In addition, because inclusion required early in-hospital SGLT2 inhibitor initiation according to routine clinical practice, treatment-selection bias related to clinician-directed therapy initiation cannot be excluded. Because the analytic cohort was restricted to patients who survived to paired 6-month reassessment, survivor bias cannot be excluded and the observed recovery trajectories may not fully represent the broader population of hospitalized patients with first-presentation HFrEF. Several limitations warrant consideration. First, this was an observational real-world cohort, and residual confounding related to incompletely characterized background therapy intensity and clinical heterogeneity cannot be excluded. Time-updated treatment variables, including GDMT titration, loop diuretic adjustment, and statin intensity, were not systematically modeled. Second, survivor bias may have influenced the observed recovery trajectories because the analytic cohort was restricted to patients surviving to paired 6-month reassessment. Third, etiologic classification reflected routine clinical assessment rather than a standardized multimodality imaging protocol, and residual etiologic misclassification therefore remains possible. Finally, several clinically relevant variables, including rhythm status, atrial fibrillation burden, revascularization status, and CKD severity, were not comprehensively characterized.

The practical implication is not that SGLT2 inhibitors should be targeted differently by etiology since current evidence supports their use across eligible patients, but rather that post-discharge expectations, imaging reassessment, and renal surveillance may need to be calibrated differently in ICM and NICM. This interpretation remains consistent with large randomized trials such as DAPA-HF and EMPEROR-Reduced, which demonstrated broad benefit of SGLT2 inhibition across diverse HFrEF populations.

The present findings should therefore be interpreted primarily as exploratory and hypothesis-generating, while supporting the concept that underlying HF substrate may shape early cardiorenal recovery after SGLT2 inhibitor initiation.

## 5. Conclusions

In this prospective real-world cohort of patients with first-presentation HFrEF who initiated SGLT2 inhibitors during hospitalization, HF etiology was associated with different 6-month cardiorenal recovery patterns. Compared with ICM, NICM showed greater improvement in LVEF and a more favorable net eGFR trajectory, whereas NT-proBNP decline was similar across groups. Within this survivor cohort with paired follow-up, these findings support the concept that early recovery after SGLT2 inhibitor initiation may remain shaped by underlying disease substrate rather than demonstrating differential efficacy according to etiology. Larger studies with standardized imaging, longitudinal treatment characterization, and more granular follow-up data are needed to further clarify etiology-associated recovery trajectories.

## Figures and Tables

**Table 1:** Selected baseline characteristics of the paired 6-month analytic cohort by heart failure etiology.

Characteristic	ICM (*n* = 90)	NICM (*n* = 151)	*p* Value
Age, years	64.03 ± 11.17	60.40 ± 14.01	0.026
NYHA III at admission, %	44.4	50.9	0.716
Arterial hypertension, %	80.7	65.1	0.006
Active smoking, %	52.6	35.8	0.010
Hyperlipidemia, %	84.4	50.9	<0.001
LVEF, % (median [IQR])	30.0 [25.0–40.0]	30.0 [22.0–35.0]	0.006
Admission glucose, mmol/L (median [IQR])	7.25 [6.00–10.90]	6.40 [5.20–7.40]	<0.001
eGFR, mL/min/1.73 m^2^	73.68 ± 21.52	75.85 ± 21.21	0.435
NT-proBNP, pg/mL (median [IQR])	4891.5 [2112–8439]	3618.5 [1779–8327]	0.289
HbA1c, % (median [IQR])	6.10 [5.70–7.05]	6.00 [5.60–6.60]	0.073
LDL cholesterol, mmol/L (median [IQR])	3.20 [2.60–4.10]	2.80 [2.00–3.60]	0.002

Baseline demographic, clinical, echocardiographic, renal, and metabolic characteristics of the paired 6-month analytic cohort stratified according to heart failure etiology. Continuous variables are presented as mean ± standard deviation (SD) or median (interquartile range [IQR]), as appropriate, and categorical variables as percentages. NYHA: New York Heart Association; LVEF: left ventricular ejection fraction; eGFR: estimated glomerular filtration rate; NT-proBNP: N-terminal pro-B-type natriuretic peptide; HbA1c: glycated hemoglobin; LDL: low-density lipoprotein.

**Table 2:** Unadjusted paired 6-month between-group comparison of change in key outcomes by heart failure etiology.

Parameter	ICM	NICM	*p* Value (Between-Group)
ΔLVEF, % (median [IQR])	+5 [0–12]	+10 [0–18]	0.049
ΔeGFR, mL/min/1.73 m^2^ (median [IQR])	−2.70 [−12.60 to 3.40]	0.30 [−5.90 to 6.60]	0.038
ΔNT-proBNP, pg/mL (median [IQR])	−2820.5 [−5595 to −907.5]	−2592 [−5878 to −1045]	0.845

Unadjusted between-group comparison of paired 6-month changes in key cardiorenal outcomes according to heart failure etiology. Δ values represent 6-month minus baseline change scores, with negative values indicating reductions from baseline. Analyses were performed using paired available-case data within the survivor cohort. eGFR: estimated glomerular filtration rate; LVEF: left ventricular ejection fraction; NT-proBNP: N-terminal pro-B-type natriuretic peptide.

**Table 3:** Baseline, 6-month, and paired change values of key cardiorenal biomarkers according to heart failure etiology.

Parameter	Timepoint	ICM	NICM	*p* Value
LVEF, %	Baseline	30.0 [25.0–40.0]	30.0 [22.0–35.0]	0.006
	6 months	40.0 [35.0–45.0]	38.0 [30.0–45.0]	0.885
	Δ change	+5 [0–12]	+10 [0–18]	0.049
eGFR, mL/min/1.73 m^2^	Baseline	73.68 ± 21.52	75.85 ± 21.21	0.435
	6 months	69.50 [54.50–88.70]	77.00 [61.00–95.60]	0.065
	Δ change	−2.70 [−12.60 to 3.40]	0.30 [−5.90 to 6.60]	0.038
NT-proBNP, pg/mL	Baseline	4891.5 [2112–8439]	3618.5 [1779–8327]	0.289
	6 months	774 [351–1792]	556 [204–1448]	0.034
	Δ change	−2820.5 [−5595 to −907.5]	−2592 [−5878 to −1045]	0.845

Baseline, 6-month follow-up, and paired change values of key cardiorenal biomarkers according to heart failure etiology. Continuous variables are presented as mean ± standard deviation (SD) for normally distributed variables and as median (interquartile range [IQR]) for non-normally distributed variables, as appropriate based on distribution testing. Δ values indicate 6-month minus baseline change scores. eGFR: estimated glomerular filtration rate; LVEF: left ventricular ejection fraction; NT-proBNP: N-terminal pro-B-type natriuretic peptide.

## Data Availability

The data are not publicly available due to restrictions related to patient privacy and in accordance with GDPR regulations. Data may be available from the corresponding author upon reasonable request and approval by the institutional ethics committee.

## Abbreviations

BMI	body mass index
CAD	coronary artery disease
CMR	cardiac magnetic resonance
eGFR	estimated glomerular filtration rate
GDMT	guideline-directed medical therapy
HbA1c	glycated hemoglobin
HF	heart failure
HFrEF	heart failure with reduced ejection fraction
ICM	ischemic cardiomyopathy
IQR	interquartile range
LVEF	left ventricular ejection fraction
NICM	non-ischemic cardiomyopathy
NT-proBNP	N-terminal pro-B-type natriuretic peptide
NYHA	New York Heart Association
SGLT2 inhibitor	sodium–glucose cotransporter 2 inhibitor
TTE	transthoracic echocardiography
